# Revascularização do Miocárdio Guiada pela Fisiologia: Está na Hora do Cirurgião Cardíaco Incorporar a Reserva de Fluxo Fracionada na Prática?

**DOI:** 10.36660/abc.20210921

**Published:** 2021-11-22

**Authors:** Tannas Jatene, Fabrício Ribeiro Las Casas, Rogerio Lobo de Andrade Las Casas, Vinicius Daher Vaz, Alberto de Almeida Las Casas

**Affiliations:** 1 Hospital do Coração Anis Rassi Goiânia GO Brasil Hospital do Coração Anis Rassi, Goiânia, GO – Brasil; 2 Hospital Israelita Albert Einstein Goiânia GO Brasil Hospital Israelita Albert Einstein, Goiânia, GO – Brasil

**Keywords:** Angiografia Coronariana/fisiologia, Doença da Artéria Coronariana/fisiopatologia, Ponte de Artéria Coronariana, Reserva Fraccionada de Fluxo Miocárdio

De acordo com as diretrizes mais recentes de revascularização do miocárdio, a intervenção coronária percutânea guiada pela reserva de fluxo fracionada (RFF) deve ser considerada em pacientes com doença de múltiplos vasos. Tal medida inclui a avaliação de todas as lesões com estenose de 40 a 90% de diâmetro antes do implante de *stent*.^1,2^ As mesmas diretrizes sugerem priorizar a revascularização completa, ou seja, em que todos os vasos com estenose superior a 50% de diâmetro devem ser tratados, quando se decide pela cirurgia de revascularização miocárdica (CRM) (*bypass*).^2^

Nesta edição dos Arquivos Brasileiros de Cardiologia, Martins et al.^3^ abordaram esse paradoxo com uma metanálise de cinco estudos, incluindo um total de 1114 pacientes, comparando a CRM guiada pela fisiologia com a CRM convencional guiada por angiografia. Embora a análise dos dados agrupados mostrou que não houve diferença nas taxas de infarto do miocárdio e revascularização do vaso alvo, observou-se uma redução de 37% no risco relativo de mortes por todas as causas com a CRM guiada pela fisiologia.

Vários estudos realizados nas duas últimas décadas revelaram desfechos favoráveis e menor custo com o uso de angioplastia guiada pela RFF, com revascularização de somente lesões funcionalmente significativas.^4-6^ Além disso, a reclassificação dos pacientes adicionando-se as informações da RFF ao escore SYNTAX melhora sua correlação com eventos após a revascularização, o que é conhecido como escore SYNTAX funcional.^7^ Todos esses dados foram traduzidos na incorporação da fisiologia invasiva à conduta na maioria dos laboratórios de cateterismo.

Nos centros cirúrgicos, no entanto, o *bypass* de lesões angiográficas acima de 50% de diâmetro é ainda o procedimento padrão. O estudo FAME (do inglês *Fractional Flow Reserve versus Angiography for Multivessel Evaluation* ou RFF versus angiografia para avaliação de múltiplos vasos) mostrou que somente 35% das lesões com 50-70% de diâmetro de estenose eram hemodinamicamente significativas,^8^ mas os cirurgiões ainda optam pelo *bypass* dessas lesões com a justificativa de prevenir contra possível progressão da aterosclerose. No entanto, estudos demonstraram que a revascularização de lesões sem relevância hemodinâmica resulta não só na falência precoce do enxerto, como na progressão mais rápida da doença arterial coronariana na artéria nativa.^9-11^ Ainda, um estudo prévio^12^ mostrou que a CRM guiada por RFF em comparação à guiada por angiografia levou a um menor número de anastomoses de enxertos e de cirurgias com circulação extracorpórea. Todos esses argumentos não têm sido suficientes para convencer os cirurgiões.

A presente metanálise contribui para essa controvérsia. Três ensaios controlados randomizados e dois estudos retrospectivos foram avaliados em conjunto. A redução na mortalidade poderia convencer os cirurgiões cardíacos a utilizar a RFF na tomada de decisão, mas algumas limitações importantes do estudo impossibilitam tal reviravolta: 1) o tamanho pequeno das amostras e consequente baixo número de eventos nos ensaios controlados randomizados; 2) o estudo com o maior número de pacientes tem delineamento retrospectivo, e assim, é sujeito a vieses; 3) a ausência de um seguimento em longo prazo, quando os possíveis benefícios da revascularização completa seriam mais evidentes; 4) a redução na mortalidade relatada por Martins et al.^3^ é difícil de ser explicada sem uma redução no infarto do miocárdio e na revascularização de vaso-alvo, e seria mais convincente se uma redução na mortalidade cardíaca também fosse revelada.

Embora o ensaio DEFER^4^ tenha mostrado uma incidência de infarto do miocárdio de apenas 2,2% em um grupo de pacientes com lesões não significativas com base da RFF após um seguimento de 15 anos, e o estudo recente ISCHEMIA tenha levantado questões sobre os benefícios de qualquer procedimento de revascularização, “colateralização cirúrgica” e “revascularização completa” ainda serão as justificativas de cirurgiões cardíacos até o desenvolvimento de um grande ensaio randomizado com um seguimento em longo prazo, comparando a CRM guiada por RFF com a CRM guiada por angiografia.

Por hora, o “heart team” deve seguir as diretrizes e utilizar a fisiologia coronária antes de se decidir sobre a necessidade de algum tipo de revascularização miocárdica. Se a decisão for pela intervenção coronária percutânea, a RFF ou índices não-hiperêmicos devem ser utilizados para guiar o procedimento. Se a decisão for pela CRM, o benefício da RFF ainda precisa ser comprovado.
