# Supplementary material for: Revascularização do Miocárdio Guiada pela Fisiologia: Está na Hora do Cirurgião Cardíaco Incorporar a Reserva de Fluxo Fracionada na Prática?
Source: Arq Bras Cardiol. 2021 Nov 22;117(6):1124–5. [Article in Portuguese] doi: 10.36660/abc.20210921 (PMC8757154; doi:10.36660/abc.20210921)
Supplement: Translation

# Translation: Physiology-guided CABG: Is it Time for Cardiac Surgeons to Incorporate FFR Into Their Practice?

**Authors:** Tannas Jatene, Fabrício Ribeiro Las Casas, Rogerio Lobo de Andrade Las Casas, Vinicius Daher Vaz, Alberto de Almeida Las Casas

**Affiliations:** 1 Goiânia GO Brazil Hospital do Coração Anis Rassi, Goiânia, GO – Brazil; 2 Goiânia GO Brazil Hospital Israelita Albert Einstein, Goiânia, GO – Brazil

**Keywords:** Coronary Angiography/physiology, Coronary Artery Disease/physiopathology, Coronary Arteryry Bypass, Factional Flow Reserve Myocardial

According to most recent international guidelines on myocardial revascularization, fractional flow reserve (FFR)-guided percutaneous coronary intervention should be considered in patients with multivessel disease. That would include evaluation of all lesions between 40 to 90% diameter stenosis before implanting a stent.^1,2^ The same guidelines suggest prioritizing completeness of revascularization when the decision is made for coronary artery bypass graft surgery (CABG), which means bypassing all lesions >50% diameter stenosis.^2^

In this issue of *Arquivos Brasileiros de Cardiologia*, Martins et al.,^3^ approached this paradox with a meta-analysis of five studies and 1,114 patients, comparing physiology-guided CABG and conventional angiography-guided CABG. Although the pooled meta-analysis showed no difference in myocardial infarction and target vessel revascularization rates, a 37% relative risk reduction in all-cause death was associated with physiology-guided CABG.

Multiple studies over the last two decades revealed improved outcomes and lower cost with the use of FFR-guided angioplasty, with revascularization of only functionally significant lesions.^4-6^ In addition, reclassifying patients by adding FFR information to the SYNTAX score improve its correlation with events after revascularization, the so-called functional SYNTAX score.^7^ All these robust data have been translated into incorporation of invasive physiology to the toolbox of most cath-labs.

In the operating rooms, however, bypassing angiographic stenosis above 50% diameter is still the standard. The FAME (FFR versus Angiography for Multivessel Evaluation) trial showed that only 35% of the 50-70% diameter stenosis lesions were hemodynamically significant,^8^ but surgeons still bypass those lesions with the rationale of preventing possible progression of atherosclerosis. However, it has been demonstrated that bypassing lesions not hemodynamically relevant not only results in early graft failure but also accelerate progression of coronary artery disease in the native vessel.^9-11^ Moreover, previous studies revealed the reduction in the number of graft anastomoses and lower rate of on-pump surgery with FFR-guided versus angiography-guided CABG.^12^ All these arguments have not been enough to convince surgeons.

The present meta-analysis adds to this controversy. Three randomized controlled trials and two retrospective studies were evaluated together. The reduction in mortality could possibly persuade cardiac surgeons to use FFR in their decision-making process. However, major weakness of the study prevents this turnaround: 1) the small sample sizes and the consequent low number of events in the randomized controlled trials; 2) the study with the highest number of patients had a retrospective design, hence subject to inherent biases; 3) absence of long-term follow-up, when possible benefits of complete revascularization would be more evident; 4) the reduced mortality reported by Martins et al.,^3^ is difficult to explain without reduction in myocardial infarction and target vessel revascularization, and would be more convincing if a cardiovascular mortality reduction was revealed.

Although the DEFER trial^4^ showed a myocardial infarction incidence of only 2.2% in a group of patients with non-significant lesions on the basis of FFR after a 15-year follow-up and the recent ISCHEMIA trial raised questions about the benefits of any revascularization procedure, “surgical collateralization” and “completeness of revascularization” will be the arguments of cardiac surgeons until we have a large randomized trial with long-term follow-up comparing FFR- versus angiography-guided CABG.

For the time being, the heart team should follow the guidelines and use intracoronary physiology as much as possible before deciding about the necessity of any myocardial revascularization. If the decision is for percutaneous coronary intervention, then FFR or non-hyperemic indices should be used to guide the procedure. If the decision is for CABG, FFR benefit is still to be proved.
